# Validation of the IBD-Control Questionnaire across different sociodemographic and clinical subgroups: secondary analysis of a nationwide electronic survey

**DOI:** 10.1093/ecco-jcc/jjad147

**Published:** 2023-09-14

**Authors:** Gerum G Gebeyehu, Frederick Taylor, Liz Dobson, J R Fraser Cummings, Stuart Bloom, Nicholas A Kennedy, Paul Christiansen, Keith Bodger

**Affiliations:** Department of Health Data Science, Institute of Population Health, University of Liverpool, Liverpool, UK; Department of Health Data Science, Institute of Population Health, University of Liverpool, Liverpool, UK; IBD Registry Ltd, London, UK; IBD Registry Ltd, London, UK; Department of Gastroenteroogy, University Hospitals Southampton, Southampton, UK; Department of Gastroenterology, University College London Hospitals NHS Foundation Trust, London, UK; Exeter Inflammatory Bowel Disease Research Group, University of Exeter, Exeter, UK; Department of Psychology, Institute of Population Health, University of Liverpool, Liverpool, UK; Department of Health Data Science, Institute of Population Health, University of Liverpool, Liverpool, UK; Aintree University Hospital, Liverpool University Hospital Foundation Trust, Liverpool, UK

**Keywords:** Inflammatory bowel disease, patient-reported outcome measures

## Abstract

**Background:**

The IBD-Control Questionnaire is a simple, generic measure of patient-perceived disease control used increasingly in clinical practice and research. We aimed to address knowledge gaps in its psychometric performance, to ensure that it can be used with confidence in a variety of contexts.

**Methods:**

We analysed 7341 responses to the IBD Registry COVID-19 survey, sent to 40 911 patients who completed an online self-assessment tool during the pandemic. Questions covered demographics, comorbidities, inflammatory bowel disease [IBD] sub-type, and IBD-Control Questionnaire and symptom scores [CD-PRO2 or UC-PRO2]. Psychometric properties of IBD-Control-8 were tested overall and within subgroups (Crohn’s disease [CD], ulcerative colitis [UC] and IBD unclassified; male and female; ≤65 and >65 years; number of co-morbidities; deprivation status).

**Results:**

Internal consistency was very strong overall [α: 0.84, ω: 0.89] and for each subgroup [α range: 0.81–0.85; ω: 0.86–0.90]. Construct validity was demonstrated by moderate correlation of each item with global rating [VAS] [*r*_s_ range: 0.47–0.65], strong correlation between IBD-Control-8 score and VAS [*r*_s_ = 0.74], moderate-to-strong with PRO2 scores [CD: *r*_s_ = −0.718; UC: *r*_s_ = −0.602] and significantly higher IBD-Control-8 scores for PRO2-remission vs PRO2-active, consistent across subgroups. Exploratory and confirmatory factor analyses demonstrated a two-factor model (items loading onto ‘Health-related Quality of Life’ [HRQoL] or ‘Treatment’ domains). Extensive tests for factorial invariance confirmed consistency.

**Conclusions:**

IBD-Control-8 is a psychometrically robust scale which can be used across a range of populations. It offers a quick, reliable, and valid method of assessing patient-perceived control. The construct of ‘control’ includes traditional HRQoL and a novel domain relating to treatment perception.

## Graphical Abstract

**Graphical Abstract:**
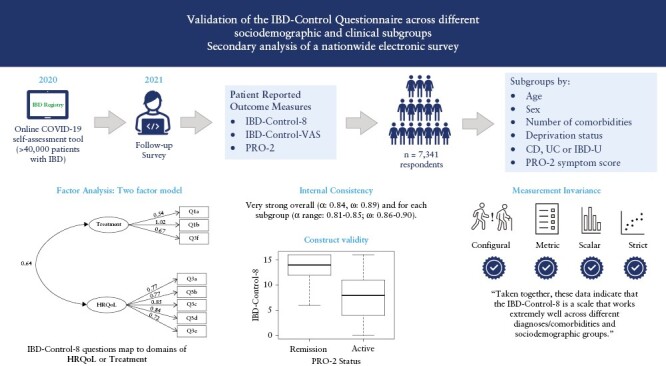

## 1. Introduction

The potential for patient-reported outcome measures [PROMs] to support patient-centred care, quality improvement, and research for inflammatory bowel disease [IBD] is increasingly recognized.^1,2^ Condition-specific PROMs vary in their outcome coverage, ranging from very short instruments [e.g. eliciting information about two dominant symptoms]^3,4^ to multi-item questionnaires (measuring the broader construct of health-related quality of life [HRQoL] which encompasses physical, social, and psychological impacts of disease).^5–7^ Multidomain instruments measuring HRQoL have been available for decades and continue to be developed, although there has been limited evidence of uptake beyond research settings.

The Inflammatory Bowel Disease Control Questionnaire [IBD-Control] was developed to measure ‘disease control from the patient perspective’.^8^ Its intended purpose was to provide a simple, intuitive screening tool for routine clinical practice. The guiding principle was to create a PROM that was generic enough to be relevant to any person living with IBD, irrespective of disease type, dominant symptoms, location, severity, or behaviour. Hence, checklists of individual gastrointestinal symptoms were excluded in favour of broad, generic items. The conceptual model for ‘disease control’ extended beyond the traditional notion of HRQoL to include patient perceptions about current treatment.^8^

The instrument’s main score is based on just eight questions [IBD-Control-8], of which five cover generic areas of HRQoL [pain or discomfort; symptoms disturbing sleep; fatigue; anxiety or depression; interference with normal activities] and three are relevant to the treatment domain. The PROM includes a visual analogue scale to elicit an overall self-rating of disease control. Originally developed in English, the IBD-Control has been independently translated into a number of other languages,^9–12^ with accumulating evidence of cross-cultural validity.^9^ Since its publication, it has achieved international endorsements^13,14^ and uptake in clinical practice,^13^ disease registries,^9,12,15^ and varied research settings.^2,16–19^ This suggests that measurement of patient-perceived control using this simple, generic tool resonates with patients, clinicians, and researchers.

The use of any PROM in such diverse contexts requires ongoing validation to ensure measurement properties are consistent. The initial validation paper^8^ did not include factor analyses exploring, and confirming, the structure of the IBD-Control scale to allow specific identification of the underlying constructs that it is measuring. Furthermore, it is critical to demonstrate that the scale structure is valid in different circumstances [e.g. during active illness compared to remission] and in different populations [e.g. across IBD subtypes, gender, age, and deprivation groups, and people with comorbidities] to ensure that it can be used with confidence by clinicians in a variety of contexts.

In the present study we aimed to expand the evidence base for the validity of the IBD-Control Questionnaire by applying factor analysis [exploratory and confirmatory], tests of factorial invariance, and measures of construct validity across different socioeconomic and clinical subgroups. Analysis of data from an online survey undertaken by the UK IBD Registry provided a unique opportunity to investigate previously unreported properties of IBD-Control using a very large, nationwide sample.

## 2. Materials and Methods

### 2.1. Data source: the IBD Registry COVID-19 follow-up survey

During the early phase of the COVID-19 pandemic, the UK IBD Registry and British Society of Gastroenterology developed and launched an online self-assessment tool to allow patients to determine risk level and support decisions about ‘shielding’.^20^ This tool used the Research Electronic Data Capture [REDCap®], a secure web-based application for building and managing online surveys.^21^ Of the patients who used the tool, 40 911 provided permission to be contacted for a follow-up survey which was hosted on the same platform. Invitations to participate in the follow-up survey were sent out in May 2021 via e-mail or SMS depending on preferences indicated when completing the tool. Reminders were sent by preferred contact method in mid-June 2021. The survey remained open to completion until the end of the study period [November 2021].

In addition to demographics [age, gender, ethnicity, place of residence], self-reported IBD diagnosis (Crohn’s disease [CD], ulcerative colitis [UC], IBD unclassified [IBD-U]), selected comorbidities [in five categories: hypertension; respiratory or chest disease; angina, heart attack, or stroke; heart failure; and heart valve disease] and current drug treatments, the survey included items relating to exposure to COVID-19; symptoms, testing and treatment for COVID-19; vaccination status; and a number of health-related outcomes. The PROMs included the IBD-Control Questionnaire^8^ and symptom-based scores.^3,4^ The electronic implementation of IBD-Control comprised the eight items required to generate the summary score [IBD-Control-8], the transitional question [same, better, worse], and the IBD-Control-VAS [visual analogue scale]. The IBD-Control-8 score ranges from 0 [worst] to 16 [best], whereas the VAS is scored 0 to 100 [0 = worst control].

Depending on self-reported diagnosis, patients were asked to complete a relevant two-symptom instrument, or ‘PRO2’. The CD-PRO2 is a subscore of the Crohn’s Disease Activity Index [CDAI] and asks about number of liquid stools per day and abdominal pain [0 = none, 1 = mild, 2 = moderate, 3 = severe] over each of the last 7 days.^4^ The 7-day average scores are summed to calculate an unweighted score. The weighted CD-PRO2 score is calculated by multiplying the 7-day averages of the number of liquid stools and abdominal pain by 2 and 5 respectively, before summing. Disease remission is defined by a weighted CD-PRO2 score <8.^4^ The UC-PRO2 is derived from the Mayo score and determines the number of stools above baseline [0 = normal number of stools, 1 = 1–2 stools above normal, 2 = 3–4 stools more than normal, 3 = 5 or more stools above normal] and rectal bleeding [0 = none, 1 = visible blood with stool less than half the time, 2 = visible blood with stool half of the time or more, 3 = passing blood alone].^3^ The scores of both items were summed to calculate a six-point scale UC-PRO-2 summary score. Disease remission was defined by a score for number of stools above baseline <2 and the absence of rectal bleeding.^3^

### 2.2. Subpopulations [strata] of interest

For the purpose of validating consistent performance properties within different sub-populations, we undertook analyses stratified by diagnosis [IBD sub-type], age [≤65 and >65 years], sex at birth, socioeconomic status [based on the Index of Multiple Deprivation, derived from area of residence and categorized into population quintiles], and the presence of other chronic conditions [number of co-morbidities].

### 2.3. Statistical and psychometric analysis

All analyses were performed within the IBD Registry’s Trusted Research Environment using de-identified data extracted from REDCap to R [Version 4.1.0, R Core Team 2021, R Foundation for Statistical Computing] using the *redcapAPI* package. Descriptive statistics for continuous variables are summarized as median [interquartile range, IQR] or mean [standard deviation, SD], as appropriate. We conducted univariate analysis using independent sample t-tests or analysis of variance [ANOVA], Mann–Whitney U test or Kruskal Wallis test, and chi-square test or Fisher’s exact test for continuous parametric, continuous non-parametric, and categorical data, respectively. Spearman’s rank correlation coefficient was used as a non-parametric measure of correlation.

Factor structure was ascertained through a two-stage process. Data were split at random [50%, 50%] into a exploratory and confirmatory data set. For exploratory factor analysis [EFA] the analysis was run on the polychoric correlation matrix rather than the raw data [due to the data being three-level ordinal]. The Kaiser–Meyer–Olkin [KMO] statistic was used to test sampling adequacy [values above 0.7 are considered good]. Bartlett’s test of sphericity was used to assess whether correlations between items were sufficiently large for exploratory factor analysis [*p* < 0.05 is indicative of sufficient correlations]. The number of factors was established using a parallel analysis, before running the exploratory factor analysis using an Oblimin rotation [as it was assumed factors would correlate]. Item loading above 0.5 and not cross-loading above 0.4 on another factor were considered valid loadings.

Following this a confirmatory factor analysis [CFA] of the structure identified by the EFA was conducted. A diagonally least squares estimator was used due to the data being ordinal.^22^ Items were free to load onto their factors, and factors were free to correlate with each another. Model fit was assessed using a range of fit indices. Two incremental fit indices were computed, the Tucker–Lewis Index [TLI] and comparative fit index [CFI], with values of above 0.90 being deemed acceptable and values of 0.95 deemed good.^23^ The absolute fit index, the root mean square error of approximation [RMSEA], was also produced where values of ≤0.05 are deemed good, values of ≤0.08 are deemed fair, values between 0.08 and.010 are deemed mediocre, and values >0.10 are considered a poor fit.^23,24^ Finally, another absolute fit index, the standardized root mean square residual [SRMR] was calculated; values <0.08 are considered a good fit.^23^ Modification indices were inspected; errors were allowed to covary if modification indices were >10, loaded on the same factor, and the correlated error terms made conceptual sense.

Internal consistency of PROM items was tested using Cronbach’s alpha and McDonald’s omega [the former being the most common method although it assumes tau equivalence with recent recommendations suggesting McDonald’s omega is a superior measure].^25^ This was conducted on the full data set, and in subgroups.

Finally, we tested measurement invariance of the scale to ensure that the factor structure and related properties were consistent across different groups. First, we tested configural invariance [whether the factor structure holds across the two samples] by fitting the factor structure identified with a grouping variable. This model was assessed using the same fit indices described for the CFA. This configural model was then compared to the metric invariance model, which was the same structure but fixing factor loadings across groups [intercepts allowed to vary]; this shows if each item contributes to the factor in a similar manner across the groups. The validity of the metric model was assessed using CFI differences [∆CFI] <0.01, RMSEA differences [∆RMSEA] <0.015, and SRMR differences [∆SRMR] <0.03 as the cut-offs for showing metric invariance. The metric invariance model was then compared to the scalar invariance model in which factor loadings and intercepts are assumed to be equal across our groups, which allows us to ascertain if we can validly compare the means of factors across groups. The assessment for this was the same as for metric invariance except the SRMR cut-off was more strict with ∆SRMR < 0.015 to indicate scalar invariance. Finally, we also tested strict invariance in which residuals as well as slopes and intercepts were assumed to be constant, thus elucidating if the item’s residual variance [i.e. unique variance] is consistent across groups. This model was compared with the metric invariance model, and the same cut-offs were used for this as in the previous model comparison.^26^

### 2.4. Ethical approval

The study was approved by the UK Health Research Authority [IRAS ID 295681] and Research Ethics Committee [REC ID 21/NI/0064]. Respondents to the survey provided electronic consent for participation.

## 3. Results

### 3.1. Demographics of survey respondents

Of 40 911 patients invited to complete the COVID-19 follow-up survey, 7341 [17.9%] returned a response of whom 7337 completed the IBD-Control-8 and IBD-Control-VAS [99.9% completion rate]. Completion rate for CD-PRO2 was 91.2% of 3808 respondents reporting a diagnosis of CD and that for UC-PRO2 was 96.8% of 3298 respondents. Baseline patient characteristics of respondents are summarized in Table 1, overall and by diagnosis. Median age of all respondents was 48 years [range: 18–92], 63% of participants were female, and 52% reported a diagnosis of CD, 45% of UC, and 3.1% of IBD-U. One-fifth of participants [19%] indicated one additional comorbidity and 5.5% reported two or more. One in 20 [5.1%] were taking prednisolone and two-thirds [67%] reported being treated with an advanced medical therapy at the time of the survey.

**Table 1.** Demographic and clinical characteristics of survey respondents and summary statistics for patient-reported outcome measures. Overall population [*n* = 7337] and stratified by self-reported diagnosis.

Characteristic	Overall	Crohn’s disease	Ulcerative colitis	IBD unclassified
Number of respondents	7337	3808	3298	231
Age, years, median [IQR]	48 [36, 58]	46 [35, 57]	49 [38, 60]	46 [34, 59]
Sex				
Female	4608 [63%]	2465 [65%]	1989 [60%]	154 [67%]
Male	2729 [37%]	1343 [35%]	1309 [40%]	77 [33%]
Deprivation status [quintile]				
1—least deprived area	2003 [27%]	1007 [27%]	934 [29%]	62 [27%]
2	1791 [25%]	908 [24%]	831 [25%]	52 [23%]
3	1524 [21%]	782 [21%]	686 [21%]	56 [24%]
4	1246 [17%]	681 [18%]	530 [16%]	35 [15%]
5—most deprived area	721 [9.9%]	401 [11%]	294 [9.0%]	26 [11%]
Place of residence				
England	6340 [87%]	3274 [87%]	2874 [88%]	192 [83%]
Wales	352 [4.8%]	181 [4.8%]	159 [4.9%]	12 [5.2%]
Scotland	502 [6.9%]	264 [7.0%]	214 [6.5%]	25 [11%]
Northern Ireland	94 [1.3%]	63 [1.7%]	29 [0.9%]	2 [0.9%]
Oral steroids [current]				
Yes	407 [5.5%]	185 [4.9%]	212 [6.5%]	10 [4.4%]
No	6869 [94%]	3591 [94%]	3061 [93%]	217 [94%]
Unsure	59 [0.8%]	30 [0.8%]	25 [0.8%]	4 [1.7%]
Advanced IBD therapies [current]	4896 [67%]	2956 [78%]	1838 [56%]	102 [46%]
Number of comorbidities				
0	5510 [75%]	5510 [75%]	2920 [77%]	2437 [74%]
1	1425 [19%]	1425 [19%]	713 [19%]	652 [20%]
>1	402 [5.5%]	402 [5.5%]	175 [4.6%]	209 [6.3%]
Patient-reported symptoms				
CD-PRO2 weighted, median [IQR]	–	6 [2, 12]	–	–
CD-PRO2 remission, *n* [%]	–	2035 [59%]	–	–
UC-PRO2 score, median [IQR]	–	–	0 [0, 1]	–
UC-PRO2 remission, *n* [%]	–	–	2151 [67%]	–
IBD-Control Questionnaire				
IBD-Control-8 score, median [IQR]	12 [8, 16]	12.0 [7.0, 15.0]	14.0 [9.0, 16.0]	12.0 [7.0, 15.5]
IBD-Control-8 Quiescent, *n* [%]	3614 [49%]	1637 [43%]	1872 [57%]	105 [45%]
IBD-Control-VAS, median [IQR]	80 [61, 94]	80 [55, 90]	85 [69, 95]	78 [55, 90]
IBD-Control-VAS Quiescent, *n* [%]	3259 [44%]	1504 [39%]	1662 [50%]	93 [40%]

### 3.2. Patient-reported outcome data

**Figure 1.**
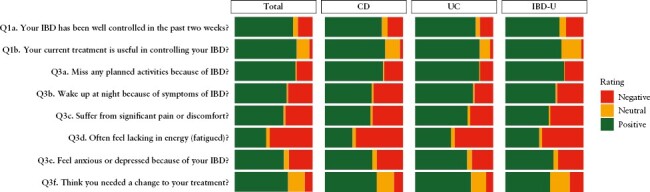
Distribution of responses to individual question items of the IBD-Control-8 among 7337 survey respondents. Overall [Total] and stratified by diagnosis (Crohn’s disease [CD], *n* = 3808; ulcerative colitis [UC], *n* = 3298; inflammatory bowel disease unclassified [IBD-U], *n* = 231). ‘Negative’ ratings indicate an answer of ‘No’ for questions 1a and 1b, or ‘Yes’ for Questions 3a to 3f, which are scored as zero in the summary score. Conversely, ‘Positive’ ratings indicate an answer of ‘Yes’ for Questions 1a and 1b, or ‘No’ for Questions 3a to 3f, which are scored as two points in the summary score. ‘Neutral’ ratings indicate answers of ‘Not Sure’ for all questions, which are allocated a score of one point.

Figure 1 illustrates the distribution of responses to individual IBD-Control items, overall and stratified by self-reported diagnosis [see Supplementary Tables S1 and S2 for tabulated data]. Overall, one in five respondents [19%] did not think their IBD was well-controlled over the last 2 weeks, with a further 6.3% being unsure. For those individual items covering traditional HRQoL domains, one in five [21%] reported missing planned activities because of IBD in the last 2 weeks, around one in three reported waking up at night because of IBD [32%], suffering significant pain or discomfort [35%], or feeling anxious or depressed because of their condition [31%]. Just over half [55%] reported often feeling lacking in energy [fatigued]. For treatment-related questions, one in five were either unsure [16%] or did not believe [4.2%] that their current treatment was useful in controlling their IBD. One in ten [11%] thought that they needed a change to treatment and a further one in five respondents [21%] were unsure. The proportions of total respondents indicating control was better, the same or worse over the last 2 weeks were 5.9, 78, and 16%, respectively.

The median overall IBD-Control-8 summary score derived from item responses was 12.0 [range: 0–16] and that of the stand-alone IBD-Control-VAS was 80 [range: 0–100]. Median scores were comparable for CD and UC. Based on the published cut-off score for ‘quiescent’ disease [IBD-Control-8 ≥ 13], 3614 [49.3%] of the total cohort would be thus classified. Corresponding figures for CD and UC were 43 and 57%, respectively.

For patients with CD, the median weighted CD-PRO2 score was 6 [range: 0–72], with 58.6% categorized as remission over the last week [score of ≤7]. For those with UC, the median UC-PRO2 score was zero [range: 0–6] with 66.9% classified as remission over the last 3 days [i.e. rectal bleeding absent and number of stools less than two above baseline per day]. However, there were 449 patients [6.19%] who reported the ‘worst possible’ response for each of their two PRO-2 symptom items—i.e. had the highest possible score on the relevant PRO-2 scale.

### 3.3. Internal consistency of IBD-Control-8 items

Measures of internal consistency for the IBD-Control-8 were very strong overall [α = 0.84, ω = 0.89; *n* = 7337 patients], consistent with originally reported findings [α=0.85, *n* = 299 patients].^8^ Uniquely in the present study, we confirmed that consistency was maintained within sub-groups of patients categorized by diagnosis, sex at birth, age groups, and deprivation status [Table 2: α and ω values consistently above 0.80; α ranging from 0.81 to 0.85; ω ranging from 0.86 to 0.90].

**Table 2.** Internal consistency of the IBD-Control-8 scale, overall and by sub-groups

Group	Cronbach’s alpha [α]	McDonald’s omega [ω] Total	McDonald’s omega [ω] Hierarchical
Overall	0.84	0.89	0.72
By diagnosis			
CD	0.84	0.89	0.71
UC	0.84	0.89	0.72
IBD-U	0.85	0.90	0.69
By gender			
Male	0.83	0.88	0.71
Female	0.84	0.89	0.72
By age group			
≥65 years	0.81	0.86	0.67
<65 years	0.84	0.89	0.72
By deprivation index			
1 and 2	0.83	0.89	0.72
4 and 5	0.85	0.90	0.73
By number of comorbidities			
No comorbidities	0.84	0.89	0.72
≥1 comorbidity	0.85	0.89	0.73

### 3.4. Construct validity of individual IBD-Control-8 items vs VAS

**Figure 2.**
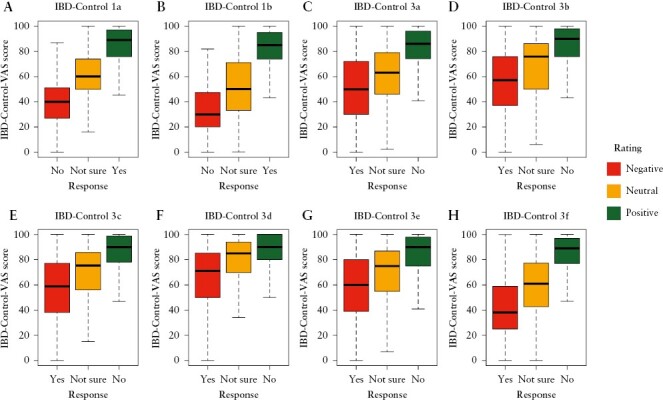
IBD-Control-VAS scores according to responses to each of the IBD-Control-8 items. [A] IBD-Control 1a. Spearman’s *R* [*R*_s_] = 0.647, *p* < 0.001; [B] IBD-Control 1b. *R*_s_ = 0.487, *p* < 0.001; [C] IBD-Control 3a. *R*_s_ = 0.472, *p* < 0.001; [D] IBD-Control 3b. *R*_s_ = 0.527, *p* < 0.001; [E] IBD-Control 3c. *R*_s_ = 0.553, *p* < 0.001; [F] IBD-Control 3d. *R*_s_ = 0.455, *p* < 0.001; [G] IBD-Control 3e. *R*_s_ = 0.475, *p* < 0.001; [H] IBD-Control 3f. *R*_s_ = 0.576, *p* < 0.001. Median IBD-Control-VAS scores were significantly different between the three response options for each of the eight items of IBD-Control [*p* < 0.001, Kruskal–Wallis].

The IBD-Control-VAS score is a surrogate global measure of the construct of interest, providing a numerical rating of patient-perceived control using a ‘feeling thermometer’ approach. If the eight questions within the IBD-Control-8 are each measuring an element of the same concept, their scored responses should correlate significantly with the IBD-Control-VAS. These hypothesized relationships were confirmed [Figure 2], with each item demonstrating significant, moderate-to-strong correlations with the VAS. As expected, the strongest correlation was observed for the most global question item [Q1a, *r*_s_ = 0.647, *p* < 0.001] as opposed to the remaining seven items [*r*_s_ values ranging from 0.472 to 0.576, all *p* values <0.001]. These analyses were consistent across sub-groups by sex, socioeconomic status, age group, and comorbidity status.

Figure 2 also confirms that responses to each question behave as a three-item ordinal Likert scale, with median scores for patients recording ‘Not Sure’ consistently lying intermediate between those responding ‘Yes’ or ‘No’. The latter finding is consistent with the simple scoring system for IBD-Control-8 items [which assigns one point for ‘Not Sure’ and either zero or two points for definitive replies]. The assumption of ordinal scaling of the three response options justifies our approach to constructing correlation matrices for factor analyses.

### 3.5. Construct validity of IBD-Control-8 summary score vs VAS

**Figure 3.**
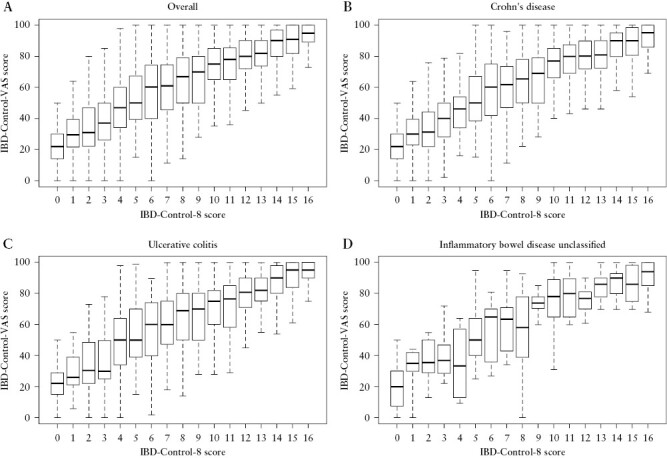
IBD-Control-VAS scores according to IBD-Control-8 score, overall and by diagnosis: [A] Overall. Spearman’s *R*_s_ = 0.738, *p* < 0.001; Kruskal–Wallis: *p* < 0.001. [B] Crohn’s disease. *R*_s_ = 0.747, *p* < 0.001; Kruskal–Wallis: *p* < 0.001. [C] Ulcerative colitis. *R*_s_ = 0.714, *p* < 0.001; Kruskal–Wallis: *p* < 0.001 [D] IBD-U. *R*_s_ = 0.745, *p* < 0.001; Kruskal–Wallis: *p* < 0.001. Boxplots show median [bold bar], interquartile range [shaded box], and range [error bars] of IBD-Control-VAS scores.

As expected, there was a strong correlation overall between the IBD-Control-8 score and IBD-Control-VAS [*r*_s_ = 0.738, *p* < 0.001; Figure 3] and this was consistent across diagnoses [CD: *r*_s_ = 0.747, *p* < 0.001; UC: *r*_s_ = 0.714, *p* < 0.001; IBD-U: *r*_s_ = 0.745, *p* < 0.001]. This relationship also held for both sexes [males: *r*_s_ = 0.701, *p* < 0.001; females: *r*_s_ = 0.750, *p* < 0.001], across age groups [age <65 years: *r*_s_ = 0.745, *p* < 0.001; age ≥65 years: *r*_s_ = 0.667, *p* < 0.001], socioeconomic groups [Deprivation Quintile 4 and 5: *r*_s_ = 0.759, *p* < 0.001; Deprivation Quintile 1 and 2: *r*_s_ = 0.714, *p* < 0.001], and for patients categorized according to number of additional co-morbidities [none: *r*_s_ = 0.733, *p* < 0.001; one or more: *r*_s_ = 0.748, *p* < 0.001].

### 3.6. Construct validity of IBD-Control-8 summary score vs PRO-2 symptom scores

**Figure 4.**
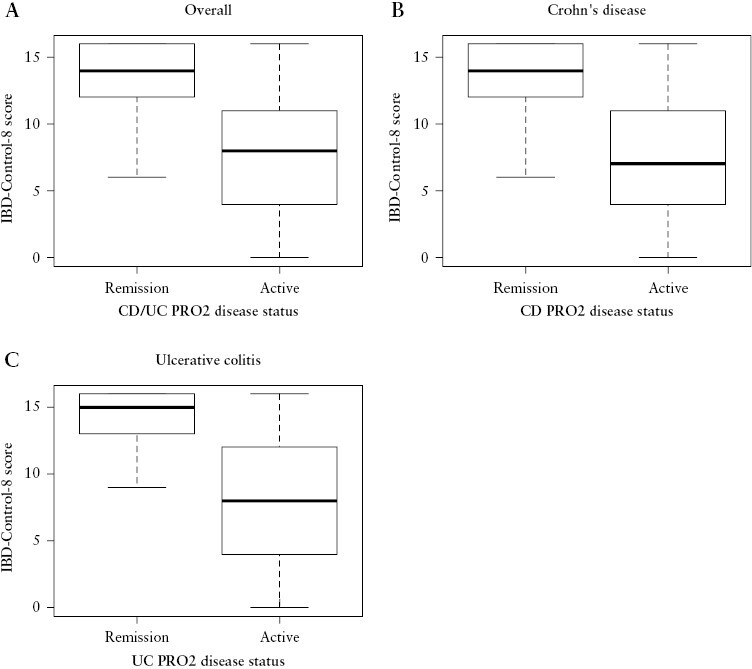
IBD-Control-8 scores according to remission status, defined using cut-offs for symptom-based scores [PRO2]. [A] Overall, Wilcoxon: *p* < 0.001; [B] Crohn’s disease, Wilcoxon: *p* < 0.001; [C] ulcerative colitis, Wilcoxon: *p* < 0.001. These data provide evidence of known-groups construct validity.

The IBD-Control-8 summary score demonstrated a moderately strong correlation with the relevant PRO-2 score for each main form of IBD [CD: *r*_s_ = −0.718, *p* < 0.001; UC: *r*_s_ = −0.602, *p* < 0.001]. The strength of these significant correlations was comparable by sex, socioeconomic groups, age groups, and according to co-morbidity status (*r*_s_ values ranging from −0.665 to −0.739 [*p* < 0.001] for CD; −0.537 to −0.640 [*p* < 0.001] for UC). Furthermore, known-group comparisons confirmed significantly higher IBD-Control-8 scores in cases categorized as in remission, as compared to active disease, based on the relevant PRO2 cut-offs [Figure 4]. These findings remained statistically consistent in stratified analyses for each of the sociodemographic and clinical subgroups.

### 3.7. Exploratory factor analysis

**Figure 5.**
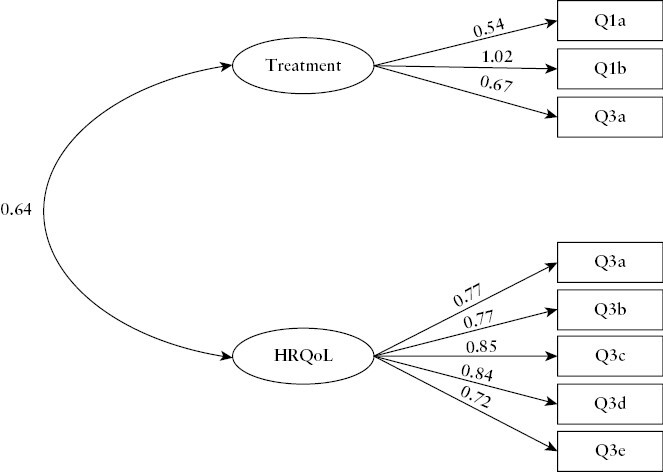
Exploratory factor analysis [EFA] confirming a two-factor representation for the measurement construct of IBD-Control-8. The eight items load to either a ‘Treatment’ factor or to a second factor representing the traditional notion of health-related quality of life [HRQoL]. Values are factor loadings which represent standardized regression coefficients.

The EFA data set had correlations between items that were sufficient for the EFA [Bartlett’s test of sphericity: *p* < 0.001], and the Kaiser–Meyer–Olkin measure of sampling adequacy was good [KMO = 0.89]. Parallel analysis suggested the data had a two-factor structure. EFA found that items in the IBD-Control-8 mapped into two factors as follows: items relating to Treatment—Q1a, Q1b, and Q3f; items relating to HRQoL—Q3a, Q3b, Q3c, Q3d and Q3e [Figure 5]. Both factors had Eigenvalues >1 [i.e. above Kaiser’s rule cut-off for valid factors]. Factor loadings are summarized in Table 3. Of note, Q1a had a cross-loading of 0.42; this is a general item asking about patient-perceived control, such that its cross-loading is consistent with the conceptual model of ‘control’ encompassing two discrete factors. We left this item to load onto factor 1 in the CFA, since any impact on fit would be identified using modification indices.

**Table 3.** Factor loadings for IBD-Control-8 question items.

Question item	Factor 1	Factor 2
Q1a. Your IBD has been well controlled in the past 2 weeks?	0.42	**0.54**
Q1b. Your current treatment is useful in controlling your IBD?	−0.07	**1.02**
Q3a. Miss any planned activities because of IBD?	**0.77**	0.07
Q3b. Wake up at night because of symptoms of IBD?	**0.77**	0.05
Q3c. Suffer from significant pain or discomfort?	**0.85**	0.02
Q3d. Often feel lacking in energy [fatigued]?	**0.84**	-0.11
Q3e. Feel anxious or depressed because of your IBD?	**0.72**	0.05
Q3f. Think you needed a change to your treatment?	0.24	**0.67**

Data represent the regression coefficients. Bold values indicate which factor each item loads onto.

### 3.8. Confirmatory factor analysis

CFA was conducted using the factor structure identified above. The initial CFA model showed a good fit of the data (CFI = 0.996, TLI = 0.995, RMSEA = 0.045 [90% CI: 0.039–0.052], SRMR = 0.038). Modification indices [MIs] showed that covariance should be added between two pairs of residuals within the domain of treatment [MI ≥ 10 between Q1b and Q3f and between Q1a and Q3f]. Model fit improved when covariance was added (CFI = 0.999, TLI = 0.998, RMSEA = 0.025 [90% CI: 0.018–0.032], SRMR = 0.023).

These data provide strong empirical evidence that the overall measurement construct encompasses questions mapping to two sub-domains, namely HRQoL and treatment.

### 3.9. Measurement invariance

The IBD-Control-8 scale showed configural invariance across all groups with good model fit in all cases, suggesting that the factor structure remains consistent across IBD-subtypes, sociodemographics, and in those with or without comorbidities [Table 4]. Likewise, Table 5 shows that there was metric invariance, which confirms that the scale items contribute to the factors in a similar way across these population strata. In addition, evidence for scalar invariance across groups was strong [Table 5], suggesting mean scores can be validly compared between subgroups. Finally, there was also strong evidence for strict invariance meaning that even the residuals were largely consistent across groups [Table 5]. The only measure that did not show substantive evidence for scalar invariance was the ∆CFI for gender being borderline [0.01], although both the SRMR and RMSEA values were well below the cut-offs for gender. Taken together, these data indicate that the IBD-Control-8 is a scale that works extremely well across different diagnoses/comorbidities and sociodemographic groups.

**Table 4.** Configural invariance model fit indices across different groups.

Grouped by:	RMSEA	CFI	TLI	SRMR
Diagnosis	0.038	0.99	0.99	0.033
Gender	0.038	0.99	0.99	0.032
Age category	0.039	0.99	0.99	0.032
Comorbidities	0.039	0.99	0.99	0.032
Deprivation	0.036	0.99	0.99	0.031

RMSEA = root mean square error of approximation; CFI = comparative fit index; TLI = Tucker–Lewis index SRMR = standardized root mean square residual; Comorbidities = number of comorbidities; Deprivation = deprivation index category [Quintile].

**Table 5.** Metric, scalar, and strict invariance across groups

	Metric			Scalar			Strict		
Grouped by:	∆RMSEA	∆CFI	∆SRMR	∆RMSEA	∆CFI	∆SRMR	∆RMSEA	∆CFI	∆SRMR
Diagnosis	−0.001	−0.001	0.001	−0.001	−0.001	0.002	0.01	−0.008	0.009
Gender	0.004	−0.003	0.004	0.001	−0.002	0.003	0.011	−0.010	0.011
Age category	0.002	−0.002	0.003	−0.002	0.000	0.001	0.003	−0.003	0.007
Comorbidities	−0.002	0.000	0.001	−0.001	0.000	0.001	0.000	−0.001	0.001
Deprivation	0.003	−0.002	0.003	−0.002	0.000	0.000	0.010	−0.007	0.009

RMSEA = Root mean square error of approximation; CFI = Comparative fit index; SRMR = Standardized Root Mean Square Residual; Comorbidities = number of comorbidities; deprivation = Deprivation index category [Quintile].

## 4. Discussion

This is the largest validation study for the IBD-Control Questionnaire reported to date and provides important new insights into its psychometric properties. Factor analysis confirms that the IBD-Control-8 has a two-factor structure [HRQoL and Treatment] which remains remarkably consistent across a range of clinical and sociodemographic groups. The scale was also found to have extremely good internal reliability. We found moderate correlations between IBD-Control-8 and symptom-based PRO scores, consistent with original work demonstrating construct validity vs clinician-reported symptom-based indices, multi-item HRQoL measures, and global clinician ratings.^8^ Correlation with PRO-2 scores per se has not been reported previously, nor has consistency of construct validity when tested across different population strata. Very high correlation between IBD-Control-8 scores and PRO-2 scores was neither expected nor desirable, since the concept of patient-perceived disease control covers a much broader construct than a symptom-based index.

IBD-Control was created as a generic screening tool that would be relevant to patients with either form of IBD, unlike symptom-based activity scores which need to be tailored to either CD or UC. Given its generic nature, we were keen to confirm that measurement properties were similar between CD and UC, males and females, younger and older age groups, and between those with, or without, additional chronic conditions. Our new evidence for invariance in instrument performance extended also to deprivation status, suggesting that responses to IBD-Control-8 items were not influenced by socioeconomic factors [e.g. educational attainment or employment status]. The simple item wording and use of three-point Likert response options were deliberate design choices for IBD-Control, seeking to minimize burden of completion, scoring, and interpretation in routine settings.^8^ Taken together, our findings suggest the IBD-Control-8 is a psychometrically robust scale which can be used reliability across a range of contexts and offers a quick method of assessing patient-perceived control of IBD.

Our study is the first to use factor analysis to generate empirical validation of the original conceptual model of the questionnaire. The eight questions mapped as expected to a two-factor model comprising HRQoL and the novel domain of ‘Treatment’. Although typically characterized by researchers as a HRQoL instrument, IBD-Control is distinct from traditional questionnaires^5^ that focus only on physical, psychological, and social impacts of disease. Our findings lend credibility to the notion that patient-perceived disease control is a measurable construct encompassing more than traditional HRQoL domains. Feelings about treatment efficacy and tolerability contribute to a patient’s sense of disease control.^8^

Despite the emergence of ‘treat-to-target’^27^ and a rightful emphasis on biochemical, endoscopic, histological, and radiological measures of inflammatory activity, the case for patient-centred assessments remains strong.^1^ The lived experience of patients with IBD is not solely driven by extent or severity of inflammation, nor is holistic management confined to decisions about anti-inflammatory drug treatment. IBD-Control was not developed to satisfy the stringencies proposed by regulatory agencies to serve as an outcome measure for clinical trials underpinning anti-inflammatory drug labelling claims.^28^ Instead, it offers a simple summary score of patient-perceived health status and contains a validated set of screening questions to highlight concerns across physical, social, psychological, and treatment domains to inform patient-centric consultations and help to quantify therapeutic deficit.^13^ Our data provide reassurance of the stability of measurement properties across different population strata, which is timely given the expanding routine use of IBD-Control within ‘apps’,^29^ patient portals, quality improvement initiatives,^13^ registries,^9,12,15^ and research.^2,16–19^

Our study has some limitations. First, the participants were patients who engaged with an online self-assessment tool^20^ and replied to an electronic survey. Case mix will not be representative of the general IBD population. The higher proportion of female respondents is typical of online health-related activity.^30^ Public awareness of factors relevant to ‘shielding’ during the early pandemic may have encouraged those taking steroids or advanced therapies to use the tool, as reflected in high rates of use of these agents in the sample. By definition, online surveys will miss digitally excluded patients who lack access to, or ability to use, the internet. We did not have access to information about phenotypic classification but do not believe there is a reason why specific subgroups should have been excluded or under-represented in this survey on the basis of disease characteristics. The impact of potential sources of selection bias is largely mitigated by very large numbers [20-fold greater than typical PROM validation studies] and by the broad geographical and sociodemographic coverage. Although an electronic survey is unlikely to have included currently hospitalized cases, there were significant numbers of cases reporting ‘worst possible’ symptoms on the relevant PRO-2 score.

Second, the survey relied on self-reported, as opposed to clinician-reported, IBD diagnosis. We cannot exclude the possibility that some respondents did not have IBD. However, numbers are likely to be small given that almost all provided details of their verifiable local IBD Service [99.6%]. Third, a cross-sectional survey does not allow assessment of test–retest reliability or sensitivity to change in health status as repeated measures are needed. Evidence for such attributes for IBD-Control has been published previously.^8,12,13^

In conclusion, we have shown that the IBD-Control Questionnaire is feasible to collect electronically at a very large, national scale to capture a global measure of patient-perceived disease control. We have demonstrated for the first time that it has strong consistency and validity across sociodemographic and clinical subgroups and confirmed the expected two-factor representation of the instrument. The measurement of patient-perceived control may offer additional insights beyond traditional PROMs focused on selected symptoms^3,4^ or HRQoL alone.^5^ Further research is needed to determine whether routine measurement of self-reported disease control [alone or in combination with other PROMs or non-invasive biomarkers] can support improvements in care with respect to safety, treatment outcome, cost-effectiveness, or patient experience. Growing uptake of IBD-Control by routine clinical services,^18^ QI programmes,^13,16,19,31^ international IBD registries,^9,12,15^ and digital e-Health solutions^29^ offers future potential for diverse repositories of data to be linked for real-world outcomes research using this simple, generic but highly reliable and valid scale.

## Supplementary Material

jjad147_suppl_Supplementary_Tables

## Data Availability

Details of how to apply to use survey data held by the IBD Registry are available online [https://ibdregistry.org.uk/analysis-and-research/apply-to-use-our-data/]. For enquiries about data access, please e-mail: analysis@ibdregistry.org.uk
