# Supplementary material for: Validation of the IBD-Control Questionnaire across different sociodemographic and clinical subgroups: secondary analysis of a nationwide electronic survey
Source: J Crohns Colitis. 2023 Sep 14;18(2):275–85. doi: 10.1093/ecco-jcc/jjad147 (PMC10896631; doi:10.1093/ecco-jcc/jjad147)
Supplement: jjad147_suppl_Supplementary_Tables [file jjad147_suppl_supplementary_tables.docx]

**Supplementary Table S1 Responses to individual items of the IBD-Control-8, overall and by self-reported diagnosis (as shown in Figure 1)**

| **Question Item** | **Overall** | **Crohn's disease** | **Ulcerative colitis** | **IBD unclassified** |
| --- | --- | --- | --- | --- |
|  | n=7,337 | n=3,808 | n=3,298 | n=231 |
| **1a. Your IBD has been well controlled in the past two weeks?** |  |  |  |  |
| No | 1,382 (19%) | 759 (20%) | 572 (17%) | 51 (22%) |
| Not sure | 459 (6.3%) | 277 (7.3%) | 162 (4.9%) | 20 (8.7%) |
| Yes | 5,496 (75%) | 2,772 (73%) | 2,564 (78%) | 160 (69%) |
| **1b. Your current treatment is useful in controlling your IBD?** |  |  |  |  |
| No | 307 (4.2%) | 165 (4.3%) | 136 (4.1%) | 6 (2.6%) |
| Not sure | 1,189 (16%) | 703 (18%) | 426 (13%) | 60 (26%) |
| Yes | 5,841 (80%) | 2,940 (77%) | 2,736 (83%) | 165 (71%) |
| **2. Have your bowel symptoms been getting worse, getting better or not changed?** |  |  |  |  |
| Better | 430 (5.9%) | 186 (4.9%) | 226 (6.9%) | 18 (7.8%) |
| No change | 5,700 (78%) | 2,967 (78%) | 2,555 (77%) | 178 (77%) |
| Worse | 1,207 (16%) | 655 (17%) | 517 (16%) | 35 (15%) |
| **3a. Miss any planned activities because of IBD?** |  |  |  |  |
| Yes | 1,527 (21%) | 921 (24%) | 551 (17%) | 55 (24%) |
| Not sure | 77 (1.0%) | 47 (1.2%) | 28 (0.8%) | 2 (0.9%) |
| No | 5,733 (78%) | 2,840 (75%) | 2,719 (82%) | 174 (75%) |
| **3b. Wake up at night because of symptoms of IBD?** |  |  |  |  |
| Yes | 2,314 (32%) | 1,458 (38%) | 778 (24%) | 78 (34%) |
| Not sure | 147 (2.0%) | 75 (2.0%) | 68 (2.1%) | 4 (1.7%) |
| No | 4,876 (66%) | 2,275 (60%) | 2,452 (74%) | 149 (65%) |
| **3c. Suffer from significant pain or discomfort?** |  |  |  |  |
| Yes | 2,567 (35%) | 1,495 (39%) | 974 (30%) | 98 (42%) |
| Not sure | 212 (2.9%) | 107 (2.8%) | 101 (3.1%) | 4 (1.7%) |
| No | 4,558 (62%) | 2,206 (58%) | 2,223 (67%) | 129 (56%) |
| **3d. Often feel lacking in energy (fatigued)?** |  |  |  |  |
| Yes | 4,041 (55%) | 2,313 (61%) | 1,606 (49%) | 122 (53%) |
| Not sure | 339 (4.6%) | 159 (4.2%) | 171 (5.2%) | 9 (3.9%) |
| No | 2,957 (40%) | 1,336 (35%) | 1,521 (46%) | 100 (43%) |
| **3e. Feel anxious or depressed because of your IBD?** |  |  |  |  |
| Yes | 2,239 (31%) | 1,273 (33%) | 891 (27%) | 75 (32%) |
| Not sure | 465 (6.3%) | 227 (6.0%) | 224 (6.8%) | 14 (6.1%) |
| No | 4,633 (63%) | 2,308 (61%) | 2,183 (66%) | 142 (61%) |
| **3f. Think you needed a change to your treatment?** |  |  |  |  |
| Yes | 779 (11%) | 442 (12%) | 297 (9.0%) | 40 (17%) |
| Not sure | 1,538 (21%) | 845 (22%) | 634 (19%) | 59 (26%) |
| No | 5,020 (68%) | 2,521 (66%) | 2,367 (72%) | 132 (57%) |

**Supplementary Table S2 Responses to individual items of the IBD-Control-8 stratified by population strata**

| **Question Item** | **Sex at birth** | | **Age Group** | | **Number of comorbidities** | | **Deprivation Status** | |
| --- | --- | --- | --- | --- | --- | --- | --- | --- |
|  | **Males** | **Females** | **<65 yrs** | **≥65 yrs** | **None** | **≥1** | **Quintile 1**  Least deprived area | **Quintile 5**  Most deprived area |
|  | N=2,729 | N=4,608 | N=6,276 | N=1,065 | N=5,510 | N=1,827 | N=2,003 | N=721 |
| **1a. Your IBD has been well controlled in the past two weeks?** | | | | | | | | |
| No | 381 (14%) | 1,001 (22%) | 1,258 (20%) | 124 (12%) | 1,024 (19%) | 358 (20%) | 301 (15%) | 179 (25%) |
| Not sure | 141 (5.2%) | 318 (6.9%) | 394 (6.3%) | 65 (6.1%) | 322 (5.8%) | 137 (7.5%) | 104 (5.2%) | 63 (8.7%) |
| Yes | 2,207 (81%) | 3,289 (71%) | 4,622 (74%) | 874 (82%) | 4,164 (76%) | 1,332 (73%) | 1,598 (80%) | 479 (66%) |
| **1b. Your current treatment is useful in controlling your IBD?** | | | | | | | | |
| No | 83 (3.0%) | 224 (4.9%) | 291 (4.6%) | 16 (1.5%) | 242 (4.4%) | 65 (3.6%) | 63 (3.1%) | 48 (6.7%) |
| Not sure | 376 (14%) | 813 (18%) | 1,033 (16%) | 156 (15%) | 848 (15%) | 341 (19%) | 284 (14%) | 142 (20%) |
| Yes | 2,270 (83%) | 3,571 (77%) | 4,950 (79%) | 891 (84%) | 4,420 (80%) | 1,421 (78%) | 1,656 (83%) | 531 (74%) |
| **2. Have your bowel symptoms been getting worse, getting better or not changed?** | | | | | | | | |
| Better | 172 (6.3%) | 258 (5.6%) | 359 (5.7%) | 71 (6.7%) | 321 (5.8%) | 109 (6.0%) | 104 (5.2%) | 49 (6.8%) |
| No change | 2,242 (82%) | 3,458 (75%) | 4,823 (77%) | 878 (83%) | 4,306 (78%) | 1,394 (76%) | 1,623 (81%) | 509 (71%) |
| Worse | 315 (12%) | 892 (19%) | 1,092 (17%) | 115 (11%) | 883 (16%) | 324 (18%) | 276 (14%) | 163 (23%) |
| **3a. Miss any planned activities because of IBD?** | | | | | | | | |
| Yes | 404 (15%) | 1,123 (24%) | 1,416 (23%) | 111 (10%) | 1,124 (20%) | 403 (22%) | 329 (16%) | 223 (31%) |
| Not sure | 23 (0.8%) | 54 (1.2%) | 67 (1.1%) | 10 (0.9%) | 55 (1.0%) | 22 (1.2%) | 25 (1.2%) | 12 (1.7%) |
| No | 2,302 (84%) | 3,431 (74%) | 4,791 (76%) | 942 (89%) | 4,331 (79%) | 1,402 (77%) | 1,649 (82%) | 486 (67%) |
| **3b. Wake up at night because of symptoms of IBD?** | | | | | | | | |
| Yes | 670 (25%) | 1,644 (36%) | 2,084 (33%) | 230 (22%) | 1,658 (30%) | 656 (36%) | 510 (25%) | 323 (45%) |
| Not sure | 65 (2.4%) | 82 (1.8%) | 122 (1.9%) | 25 (2.4%) | 97 (1.8%) | 50 (2.7%) | 33 (1.6%) | 9 (1.2%) |
| No | 1,994 (73%) | 2,882 (63%) | 4,068 (65%) | 808 (76%) | 3,755 (68%) | 1,121 (61%) | 1,460 (73%) | 389 (54%) |
| **3c. Suffer from significant pain or discomfort?** | | | | | | | | |
| Yes | 691 (25%) | 1,876 (41%) | 2,314 (37%) | 253 (24%) | 1,815 (33%) | 752 (41%) | 566 (28%) | 334 (46%) |
| Not sure | 72 (2.6%) | 140 (3.0%) | 178 (2.8%) | 34 (3.2%) | 153 (2.8%) | 59 (3.2%) | 61 (3.0%) | 23 (3.2%) |
| No | 1,966 (72%) | 2,592 (56%) | 3,782 (60%) | 776 (73%) | 3,542 (64%) | 1,016 (56%) | 1,376 (69%) | 364 (50%) |
| **3d. Often feel lacking in energy (fatigued)?** | | | | | | | | |
| Yes | 1,170 (43%) | 2,871 (62%) | 3,617 (58%) | 424 (40%) | 2,929 (53%) | 1,112 (61%) | 946 (47%) | 491 (68%) |
| Not sure | 141 (5.2%) | 198 (4.3%) | 268 (4.3%) | 71 (6.7%) | 260 (4.7%) | 79 (4.3%) | 91 (4.5%) | 21 (2.9%) |
| No | 1,418 (52%) | 1,539 (33%) | 2,389 (38%) | 568 (53%) | 2,321 (42%) | 636 (35%) | 966 (48%) | 209 (29%) |
| **3e. Feel anxious or depressed because of your IBD?** | | | | | | | | |
| Yes | 665 (24%) | 1,574 (34%) | 2,025 (32%) | 214 (20%) | 1,627 (30%) | 612 (33%) | 493 (25%) | 301 (42%) |
| Not sure | 176 (6.4%) | 289 (6.3%) | 393 (6.3%) | 72 (6.8%) | 341 (6.2%) | 124 (6.8%) | 107 (5.3%) | 58 (8.0%) |
| No | 1,888 (69%) | 2,745 (60%) | 3,856 (61%) | 777 (73%) | 3,542 (64%) | 1,091 (60%) | 1,403 (70%) | 362 (50%) |
| **3f. Think you needed a change to your treatment?** | | | | | | | | |
| Yes | 229 (8.4%) | 550 (12%) | 715 (11%) | 64 (6.0%) | 573 (10%) | 206 (11%) | 193 (9.6%) | 104 (14%) |
| Not sure | 507 (19%) | 1,031 (22%) | 1,326 (21%) | 212 (20%) | 1,089 (20%) | 449 (25%) | 356 (18%) | 181 (25%) |
| No | 1,993 (73%) | 3,027 (66%) | 4,233 (67%) | 787 (74%) | 3,848 (70%) | 1,172 (64%) | 1,454 (73%) | 436 (60%) |
